# Measurement of Local Partial Pressure of Oxygen in the Brain Tissue under Normoxia and Epilepsy with Phosphorescence Lifetime Microscopy

**DOI:** 10.1371/journal.pone.0135536

**Published:** 2015-08-25

**Authors:** Cong Zhang, Samuel Bélanger, Philippe Pouliot, Frédéric Lesage

**Affiliations:** 1 École Polytechnique de Montréal, Department of Electrical Engineering, C.P. 6079 succ.Centre-ville, Montreal, Quebec, Canada, H3C 3A7; 2 Montreal Heart Institute, 5000 Bélanger Est, Montreal, Quebec, Canada, H1T 1C8; Royal College of Surgeons in Ireland, IRELAND

## Abstract

In this work a method for measuring brain oxygen partial pressure with confocal phosphorescence lifetime microscopy system is reported. When used in conjunction with a dendritic phosphorescent probe, Oxyphor G4, this system enabled minimally invasive measurements of oxygen partial pressure (pO2) in cerebral tissue with high spatial and temporal resolution during 4-AP induced epileptic seizures. Investigating epileptic events, we characterized the spatio-temporal distribution of the "initial dip" in pO2 near the probe injection site and along nearby arterioles. Our results reveal a correlation between the percent change in the pO2 signal during the "initial dip" and the duration of seizure-like activity, which can help localize the epileptic focus and predict the length of seizure.

## Introduction

The nature of coupling between neuronal activity and the associated metabolic response is a subject of great debate [1–3]. Evaluating tissue oxygen changes and quantifying oxidative metabolism is crucial for the understanding of neuropathologies in the brain, such as Parkinson’s disease, stroke, epilepsy, Alzheimer’s disease [4–7] and developing effective therapies. While blood oxymetry provides a proxy for tissue oxygenation, under conditions of large metabolic demand and/or non-linear hemodynamic response [8], such as in epilepsy, measuring blood oxygenation alone is not sufficient. In order to investigate such conditions, monitoring of the spatio-temporal characteristics of oxygen changes in cerebral tissue is crucial.

Several techniques have been developed to measure cerebral oxygenation *in vivo*, including positron emission tomography (PET), near-infrared spectroscopy (NIRS), blood-oxygenation level dependent functional magnetic resonance imaging (BOLD-fMRI) and oxygen polarimetric electrodes [9–12]. BOLD-fMRI and NIRS are noninvasive, while PET is minimally invasive (requires an exogenous marker), and all three are utilized widely in clinical research. However, each of these techniques has limitations. BOLD-fMRI measures oxygen consumption indirectly through a complex combination of flow, volume and deoxyhemoglobin concentration. PET provides measurements of oxygen by monitoring short-lived positron emitting radionuclides, such as ^15^O, and can thus be carried out only nearby a cyclotron. Thus, both PET and BOLD-fMRI require expensive and bulky instrumentation. NIRS, on the other hand, has the advantage of portability, low cost and excellent temporal resolution; however, it measures oxygen saturation of hemoglobin, which is a proxy of oxygen concentration in blood as opposed to partial pressure (pO_2_) in tissue. Moreover, the above techniques suffer from low spatial resolution, ranging from millimeters to centimeters. Measurements by oxygen sensitive electrodes–the gold standard of oximetry, are capable of fast assessment of pO_2_, but these are invasive by nature and confined to discrete locations.

Among optical approaches, oxygen-dependent quenching of phosphorescence stands out in its ability to provide fast absolute measurements of pO_2_, which are not affected by optical parameters of the tissue [13]. Oxygen-dependent quenching phosphorescence is an optical method for oxygen sensing in biological systems, which offers excellent specificity, high sensitivity and relative simplicity of implementation [14–16]. One implementation of the technique is based on lifetime imaging in combination with microscopy and O_2_-sensitive phosphorescent probes [17–20]. With the emergence of phosphorescent probes that are water-soluble and nontoxic [14] the spatio-temporal evolution of oxygen in tissue can be investigated in greater details. Two distinct approaches have been used to experimentally determine the phosphorescence lifetime. A time-domain approach, whereby the phosphorescent probe is excited by a light pulse, and a frequency-domain approach whereby the probe is excited continuously by sinusoidally modulated light [21]. For imaging applications, the time-domain approach has been the most common *in vivo*. Several examples of microscopic measurements of phosphorescence have been reported [18,22–24], including recent improvements using new probes tailored for multiphoton excitation [25–27].

In this work we developed a confocal system to measure pO_2_ in tissue and vessels with high transverse and axial resolution, avoiding phosphorescence signal contamination from neighboring voxels. We optimized the recording conditions to reduce the prospect of measurement errors induced by photo-consumptive effects of the probe. The system allows fast data collection, avoiding excessively long data averaging, enabling us to perform pO_2_ measurement at multiple locations. We then exploited this system to investigate tissue oxygenation in a model of epilepsy.

## Materials and Methods

### Principle of phosphorescence quenching imaging

Our methodology is based on oxygen-dependent quenching of phosphorescence of metallo-porphyrins, whereby the phosphorescence decay rate is directly related to the concentration of O_2_ molecules in the medium either *in vitro* or *in vivo*. Dynamical quenching of phosphorescence involves collisions between the quencher molecules and the probe (metalloporphyrin) in its excited triplet state, resulting in radiationless deactivation and return to the ground state. Phosphorescence quenching by O_2_ is a function of the probability of collisions between the excited state probe and molecular oxygen, which is appropriately described by the Stern-Volmer equation [13],

$$\frac{\tau_{0}}{\tau }=1+K_{q}\cdot \tau_{0}\cdot pO_{2}$$ (1)

where τ_0_ and τ represent the phosphorescence lifetimes in the absence of oxygen and at a given pO_2_, while K_q_ represents the quenching constant, both factors being dependent on the temperature. The phosphorescence decay time τ is a robust and quantitative indicator of oxygen in the environment, as it is not affected by the probe concentration, and/or absorption of light by endogenous biological chromophores, such as myoglobin, hemoglobin, or cytochromes.

### Phosphorescent probe Oxyphor G4

**Fig 1:**
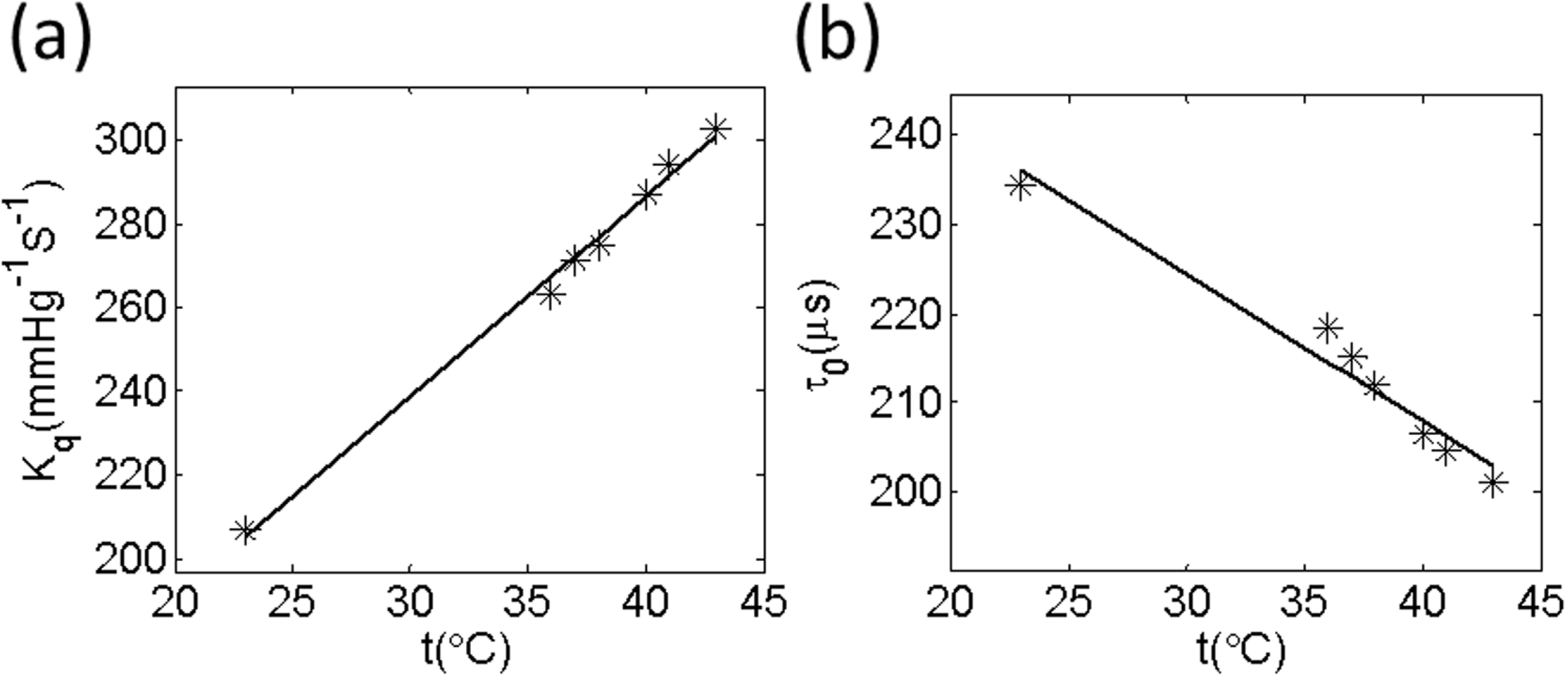
Temperature dependencies of oxygen quenching constants (Kq) and lifetimes (τ0) for G4 (a and b). The measurements were performed using 50 μM solutions of the probes, pH 7.23.

Phosphorescence lifetime microscopy has been used previously to measure pO_2_ changes through phosphorescence of exogenous probes. Early phosphorescent probes, based on Pd porphyrins [13,28], required pre-binding to a macromolecular carrier (e.g. albumin) in order to enhance their aqueous solubility and bring their quenching parameters into the range compatible with physiological oxygen concentration [13,29]. Moreover, the albumin was a potential source of toxicity. Recently, Esipova et al. [30] developed a new probe, Oxyphor G4, which is free of these limitations. Oxyphor G4 is derived from Pd-meso-tetra-(3, 5-dicarboxyphenyl)-tetrabenzoporphyrin (PdTBP) and belongs to the group of dendritic oxygen probe [31]. It is highly soluble in aqueous environments and does not permeate biological membranes. It can operate in either albumin-rich (blood plasma) or albumin-free (interstitial space) environments at all physiological oxygen concentrations, from normoxic to deep hypoxic conditions. Oxyphor G4 used in these studies was obtained from Oxygen Enterprises Ltd (University of Pennsylvania, Philadelphia, PA 19104–6059, USA). Received Oxyphor G4 was calibrated before the experiments, first equilibrated with room air (21% O_2_) and then with a completely deoxygenated solution at various temperatures (results of calibration are shown in Fig 1). In vivo the measured parameters (K_q_ and τ_0_) were selected at temperature ~37°C. The phosphorescence lifetimes of Oxyphor G4 range from ~23 to ~215 μs in the physiological pO_2_ range (160 mmHg-0 mmHg).

### Animal preparation

Animals were used according to the recommendations of the Canadian Council on Animal Care, and all procedures were approved by Animal Research Ethics Committee of the Montreal Heart Institute (Permit Number: 2013-32-01). Eight male C57BL/6 mice (8 weeks old, 20-25g weight) were anesthetized by injection of urethane (2 mg/g body weight) in a 10% (wt/vol) saline solution. Body temperature was maintained at 37°C with controlled heating blanket. Mice were ventilated via a tracheotomy using ambient air. After positioning mice on a stereotactic frame, the scalp was retracted, and 5 × 5mm sections of the skin were removed over the coronal suture around bregma (AP: -1.5 mm DV: +1.5 mm). The somatosensory cortex was exposed and the bone was removed over a region along the coronal suture closer to the bregma. Following brain exposure, 500nL of 50μM solution of Oxyphor G4 was injected in the tissue via a 34G bevelled syringe with a microsyringe pump controller (uMC4, World Precision Instruments, Sarasota, FL) over a period of 10 min. The syringe was lowered to a depth ~300μm for injections. A glass coverslip window (5mm in diameter) was then installed using agarose gel and fixed with dental acrylic cement.

For epilepsy experiments, epileptiform activity was induced in 6 mice by injecting of 500nL of the K^+^-channel blocking agent 4-AP (A78403, Sigma-Aldrich, St. Louis, MO) solution (100mM), mixed with Oxyphor G4, through a syringe pump controller, similar to the injection of Oxyphor G4 alone described above. A higher concentration of 4-AP was required in this study to generate regular seizures, mixing with G4 may reduce the potency of 4-AP thus requiring these higher dosages. Following injection, a tungsten microelectrode (0.5–2 MΩ) was placed around the glass coverslip and inserted ~500μm into the cortex with an angle ~30° (leading to a depth of 250μm) in order to record extracellular local field potential (LFP). At the end of experiments, animals were sacrificed by cervical dislocation while under anesthesia.

### Phosphorescence lifetime microscopy setup and pO_2_ estimation

**Fig 2:**
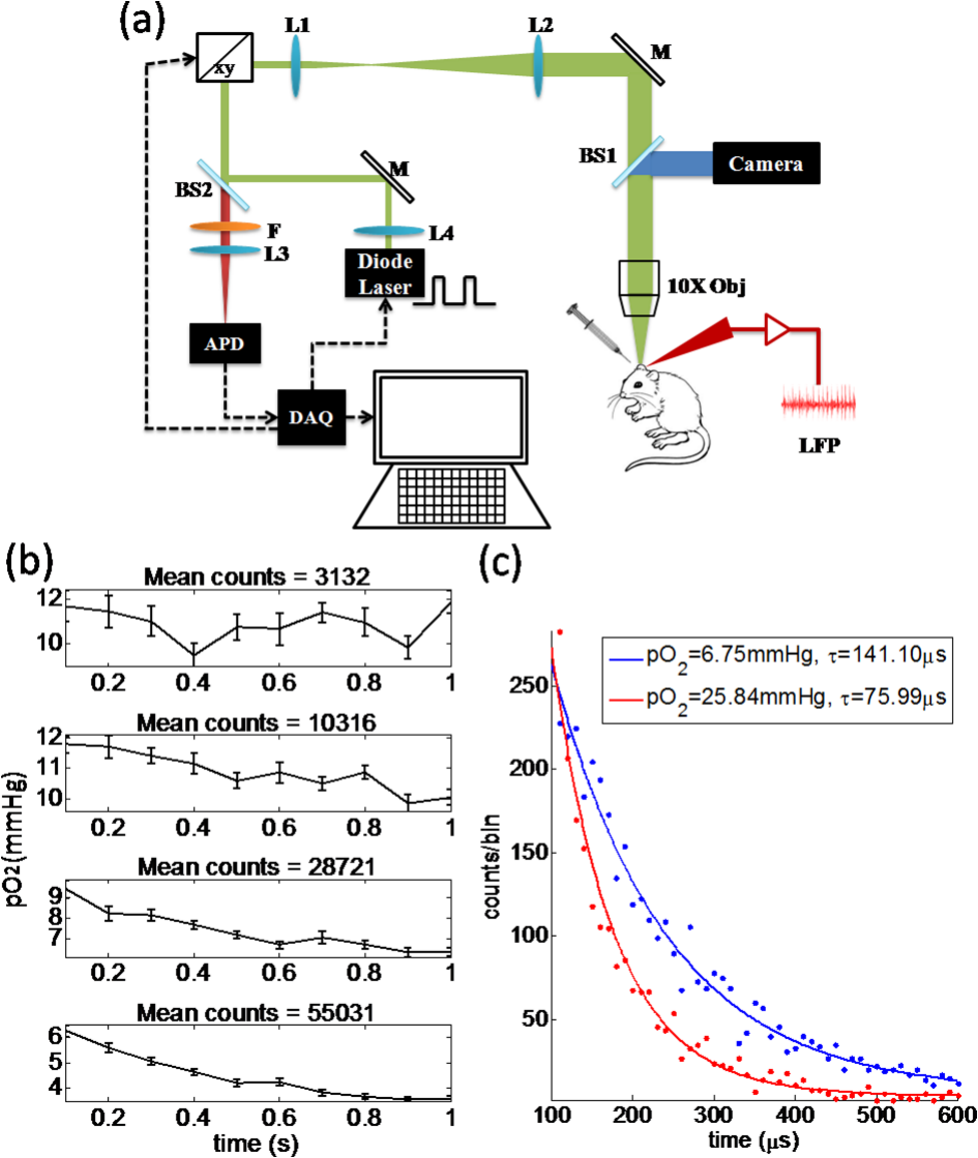
(a) Schematic of the confocal lifetime imaging system. Excitation light is provided by a laser diode (λ = 637nm, 170 mW maximum power, which is collimated by a convex lens (L1) and travels through the objective for illumination. It is focused onto the cranial window by a 10×magnification objective (Obj), which is directed to the specific points using galvanometric scanners (xy). Emitted phosphorescence light is separated from excitation light using a beam splitter (BS2) and filter (F) and detected with an avalanche photodiode (APD). The system is controlled by a computer through a data acquisition card (DAQ). (b) In vivo measurements of pO_2_vs mean counts per millisecond as controlled by the diode laser power. Higher laser powers correlate with higher consumption of O_2_ leading to a significant decrease of pO_2_ estimates over time (seen in the first point when average counts exceed 10000). When limiting to 3000 average counts, no significant decrease in pO_2_ could be measured over time. (c) Example of phosphorescence decay profiles under conditions where photo-consumption is negligible. Higher O_2_ concentration causes more quenching of phosphorescence signal, and consequently a faster decay (red profile).

The confocal lifetime system is depicted in Fig 2. The excitation light is provided by a laser diode at 637 nm (Thorlabs: HL63133DG), controlled by a data acquisition (DAQ) board (National Instruments USB-6343). A galvanometer mirror positioning system (Thorlabs: GVS002) guides the excitation beam to selected points in the focal plane with a telescope consisting of two convex lens (f_1_ = 50 mm and f_2_ = 125 mm). The response time of galvanometric scanners was 3ms. A 10×magnification objective lens (Olympus PLN10x NA = 0.25) is used to focus the light onto the sample. The phosphorescence light travels back through the telescope to be separated by a beam splitter (BS2, Semrock: FF685-DI02-25×36) and a band-pass filter (Semrock: FF02-809/81-25, 768.5 nm ~ 849.5 nm), so that only the emitted phosphorescence signal is collected by a photon-counting avalanche photodiode (APD) (Micro Photon Devices: PDM series), whose active area (50 μm in diameter) functions as a pinhole. The APD amplifier outputs a TTL pulse for each detected photon and the pulsed are counted by the DAQ board. A second beam splitter (BS1, Semrock: FF520-DI02-25×36) is placed between the objective lens and the telescope to be used in combination with a camera (Thorlabs: DCC1545M) to gather anatomic references during experiments using LED illumination. The system is controlled by a computer running custom-designed software written in Matlab (The MathWorks, Natick, MA). The software allows adjustment of the length of the excitation pulse (temporal gate), and selection of points at which pO_2_ values are measured. The in-plane resolution of the system in non-diffusive media is ~1.9 μm and axial resolution ~20.1μm. Assuming a Gaussian beam, the radius of each measured region is ~1.5 μm. In all experiments below, the excitation power of laser diode after the objective was kept below 6.5mW.

Experiments are performed in two steps. Focus is first achieved on the cortex and the animal is moved towards the objective to measure tissue at a depth of ~100 μm. Initially, a few points of interest are selected for measuring pO_2_. Survey scan phosphorescence recordings are obtained by scanning these points and summing photon counts at each point using an excitation pulse duration and phosphorescence detection window of 100 μs and 500 μs respectively. From the survey scan counts of these points, the power of the laser diode for each point is adjusted for ensuing scans to minimize the photo-oxidative damage to tissue and limit consumption according to the number of measured counts in the survey. A value of counts around 3000 was sought based on Fig 2(B). Thus following survey scans, the same points are measured while averaging 100 times to increase the signal-to-noise ratio at varying laser power. Representative excitation and decay profiles are provided in Fig 2(C). Consequently, each pO_2_ measurement at a given location required around 250 ms.

All data was processed using software custom-written in Matlab. Using a weighted least squares fitting routine, the resulting time decay curves at each point are fit with a single-exponential function:

$$I(t)=I_{0}\;\text{exp}(-\frac{t}{\tau })+c$$ (2)

where I(t) represents the light intensity at time t and I_0_ is the initial value of light intensity at time t = 0. Here τ is the phosphorescence lifetime at the point being measured and c is the magnitude of the baseline I_0_. Following the estimated parameter τ, the Stern-Volmer formula was applied to calculate the pO_2_ values.

### Normal and variable F_i_O_2_ experiments

The system was first tested in vivo by monitoring pO_2_ in cortical tissue near an artery and by varying the fraction of inspired oxygen (F_i_O_2_). In the first mouse, pO_2_ values were measured in cortical tissue near the artery during the normal atmospheric gas fraction, which the F_i_O_2_ is 21%. In a second mouse, the inspired gas fractions was changed from normal to higher F_i_O_2_ from 21% to 40% for 10 minutes at t = 260s. During each experiment, pO_2_ values were measured at multiple selected positions.

### pO_2_ values and LFP data analysis in 4-AP injected mice

The LFP data was obtained from the tungsten electrode, which was filtered by a band-pass filter between 10 and 5000Hz, amplified 1000 times with a microelectrode AC amplifier (model 1800, A-M systems, Sequim, WA), and digitized at 10kHz. In post-processing the LFP data was filtered using a Butterworth digital filter between 0.2 and 130Hz. LFP data were acquired simultaneously to pO_2_ to measure the onset-time of seizures and their duration. Since all measures were started a few minutes following 4-AP injection, identifying a baseline value pO_2_ value was difficult. Therefore, the tissue pO_2_ data were converted to percent change by subtracting then dividing the average value obtained over a 15s block of time before the onset of the epileptic events by the formula:

$$V_{\%}=(V_{raw}-V_{mean})/V_{mean}\times 100\%$$ (3)

where V_%_ is the final value of pO_2_, V_raw_ is the raw value and V_mean_ is the average value over 15s block of pO_2_ before onset. All data were expressed as means ± SE of mean (SE).

## Results

### pO_2_ in normoxia and during variable F_i_O_2_

**Fig 3:**
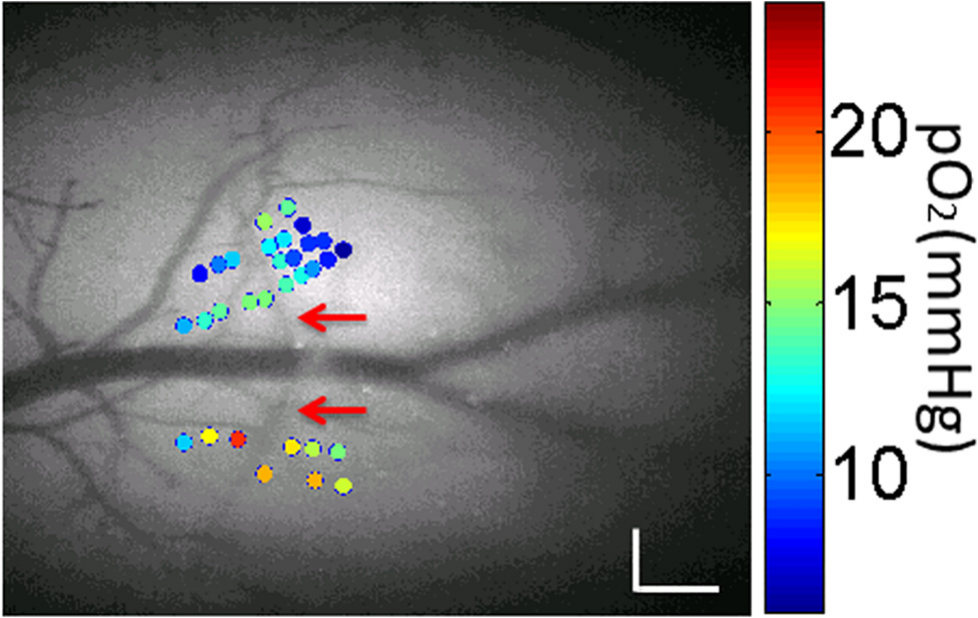
Measured pO_2_ values during normoxia (color dots), overlaid with a grayscale angiogram of cortical pial tissue from an exposed window(with an artery shown by the red arrows). The size of scale bar is 0.2mm.

The spatial pO_2_ profiles of tissue near an artery at 38 locations were measured in the somatosensory cortex of an anesthetized mouse during normoxia (Fig 3). Obtained pO_2_ values were within the range of 6-25mmHg, in concordance with previously established cortical pO_2_ levels by two-photon phosphorescence quenching technique [27]. Relatively high pO_2_ values were measured close to a large artery, with a rapid pO_2_ decrease at locations slightly further away from it reflecting values from the capillary bed. These results were in concordance with the fact that tissue pO_2_ gradients exist at the cortical surface near arteries [32].

**Fig 4:**
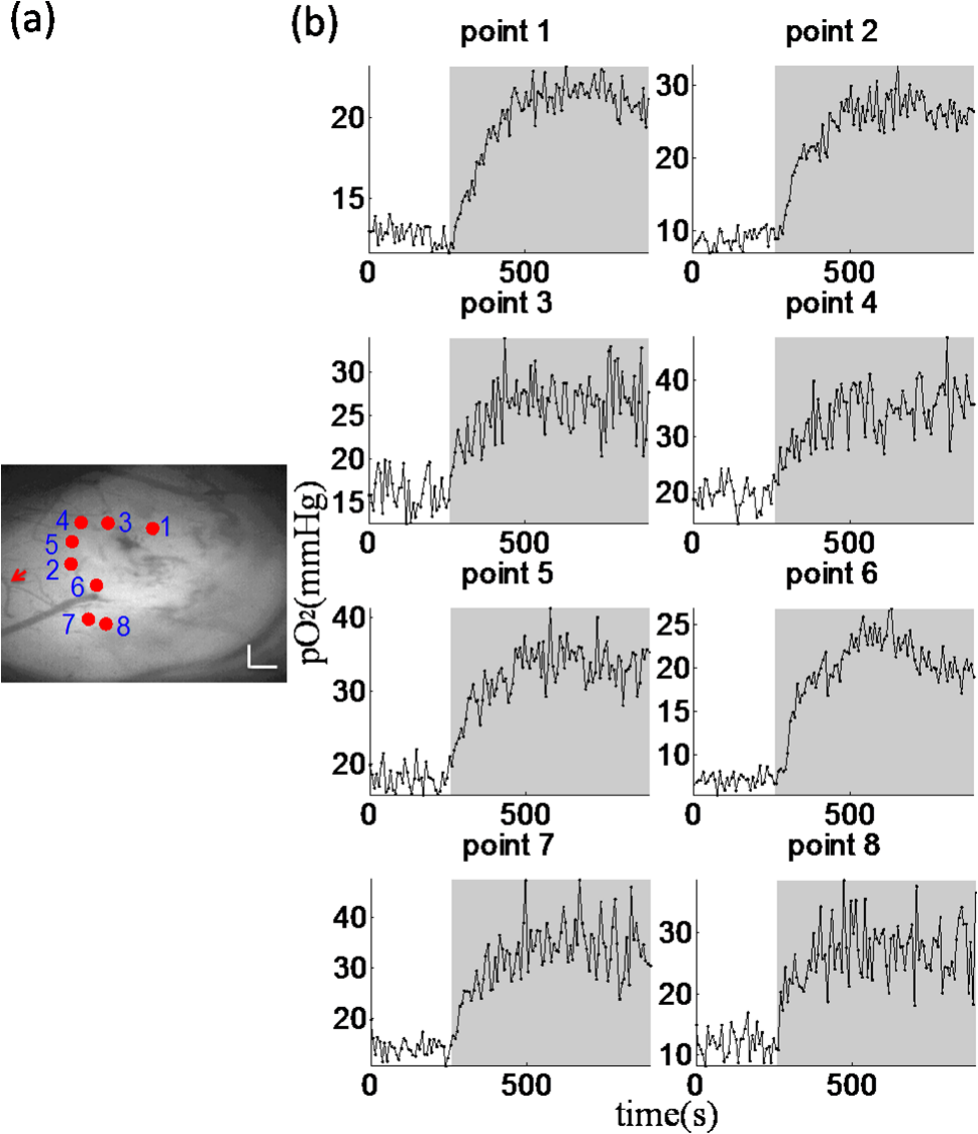
(a) Grayscale angiogram of cortical pial tissue with points of interest (red dots). Scale bar size: 0.2mm (b) Corresponding temporal profiles of pO_2_ measured while altering FiO_2_. The gray segments denote the 10 minutes period during which FiO_2_ was increased up to 40%.

Confocal lifetime measurements have the capability of simultaneously monitoring tissue pO_2_ at multiple locations. In a second animal, we obtained temporal pO_2_ profiles at selected tissue locations as the F_i_O_2_ was altered from 21% to 40% (Fig 4). During the first few minutes at 21% F_i_O_2_, the surplus O_2_ in the tissue met the metabolic demand. Our measurements (13.9±4.1 in tissue during the first few minutes) were found within the range of 5 to 25mmHg. Upon increasing the F_i_O_2_ from 21% to 40%, pO_2_ increased greatly and then saturated. An increase of 13.1 ± 3.1 mmHg in tissue from normoxic to hyperoxia was observed. Following this change in F_i_O_2_, pO_2_ values reached their peak after 191.5 ± 27.0 s in tissue.

### Tissue oxygenation at the focus and in the surrounding area during seizure-like activity

**Fig 5:**
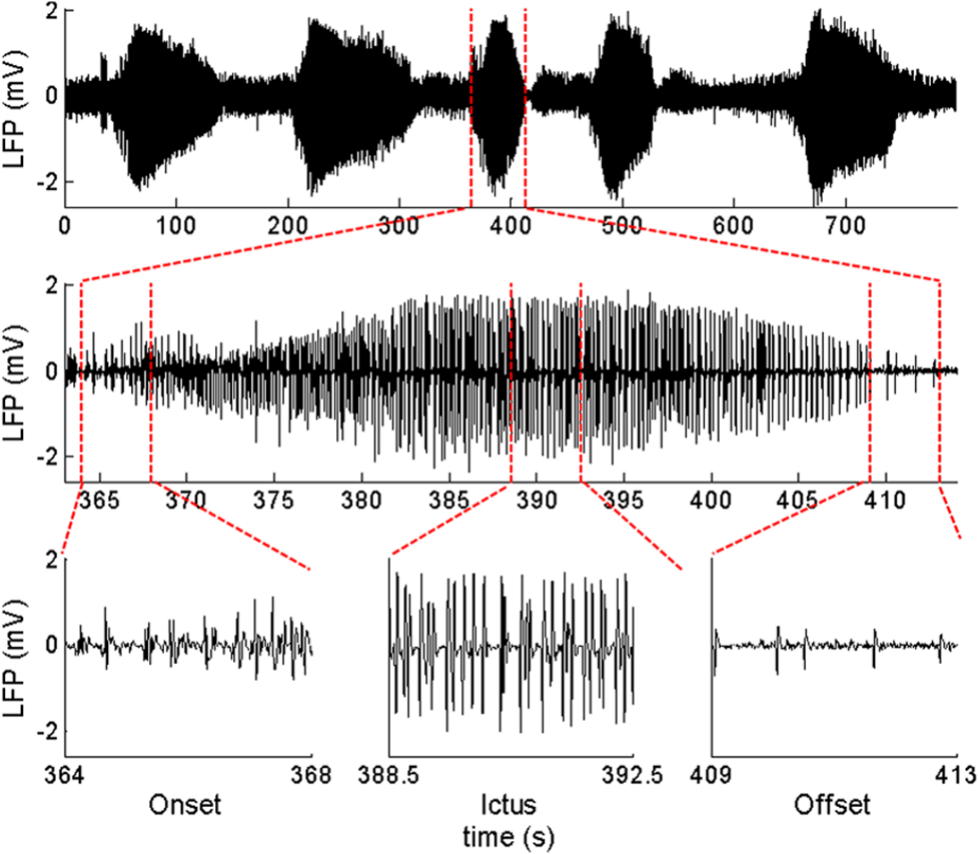
Electrophysiology of 4-AP induced epileptic activity. Top: example of ictal discharges after the 4-AP injection. Middle: zoom on an ictal discharge. Bottom: expanded view of showing the onset of the discharge, the intermediate phase and the offset.

Seizure-like activity was elicited with injection of 4-AP and recorded by local field potentials with a tungsten electrode, and were characterized by fast rhythmic spiking activity of increasing amplitude and decreasing frequency, evolving into rhythmic spikes and slow wave activity prior to gradual offset (see e.g. Fig 5). One hundred and twenty-one (121) seizure-like events were recorded in 6 mice with mean (±SE) duration of 68.9±35.1s.

**Fig 6:**
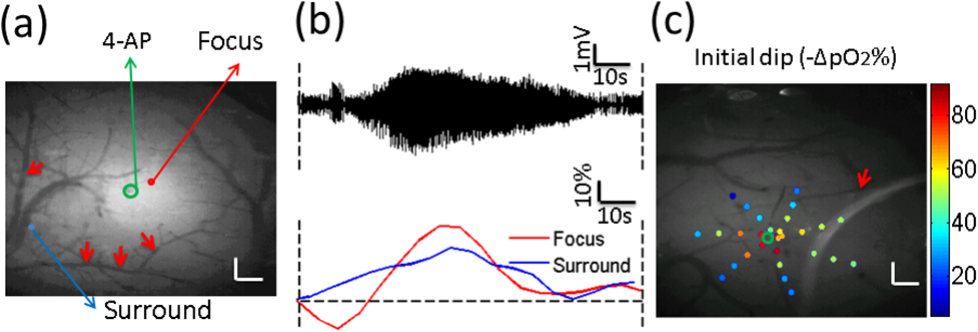
Obtained pO_2_ values in tissue near the focus and surround. (a) Grayscale angiogram of cortical surface and locations for pO_2_ measurement (red: focus; blue: surround). The artery was shown by the red arrows. Scale bar size: 0.2mm (b) Epileptic activity induced a transient dip in tissue pO_2_ followed by an increase in pO_2_ in the focus. A sustained increase in pO_2_ was seen in the surround. The dashed vertical lines show the ictal onset (left) and offset (right). (c) Distribution of percent of initial dip at multiple locations (color dotted) during epileptic activity. The 4-AP injection site is shown by green circle. The artery was shown by the red arrows. Scale bar size: 0.2mm.

To study simultaneously changes of tissue oxygenation at the focus and surrounding areas, points were selected both adjacent and distant from injection site in 3 mice (Fig 6A). An example of change in pO_2_ in the focus and surrounding areas from a single animal is shown in Fig 6B. At the focus, the typical pO_2_ profile was biphasic with an early dip after ictal onset (deoxygenation), followed by a longer duration increase in pO_2_ (hyperoxygenation). The early dip in the focus was described in previous papers during seizures [7] and was present in most seizures measured here. At a distance from the ictal focus, the pO_2_ was monophasic and significantly increased, returning to the baseline at the offset of the seizure. These results were in agreement with tissue oxygen measured by oxygen microelectrodes [33]. To assess the spatial distribution of pO_2_ around the focus, points were scanned near the focus in the form of a spiral during epileptic activity, where initial dips were measured. Fig 6C shows an example of measured percent of initial dip for different locations overlaid on a grayscale anatomical image. The higher values were obtained near the focus, and pO_2_ decreased when points were farther away from the focus. These data indicated that the influx of blood into the focus was inadequate to perfuse the hypermetabolic neurons, after which there was a period of hyperperfusion and hyperoxygenation.

### Correlation between the percent of initial dip and distance from the artery

**Fig 7:**
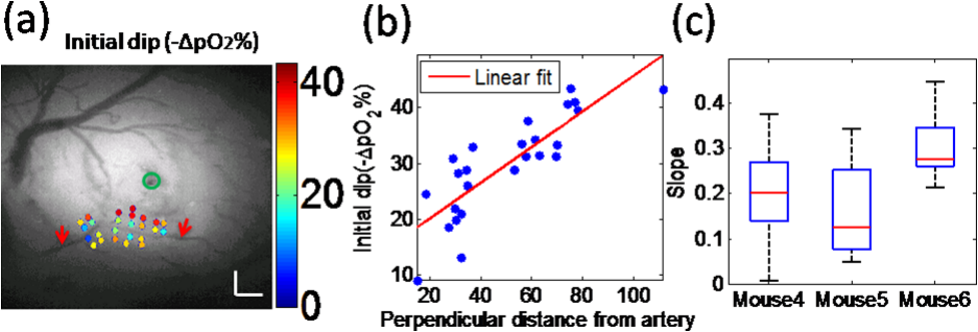
Correlation between percent change of the initial dip at multiple locations and distances from an artery (a) Measured pO_2_ values of different points near an artery during the epileptic seizure (color dots), overlaid with a grayscale anatomy (with an artery shown by the red arrows). Scale bar size: 0.2mm (b) Relationship between distance from an artery for multiple points and initial dip during one epileptic seizure. The line of linear fit is *y* = 0.32*x* + 13.7, R^2^ = 0.7182. (C) Boxplots of slopes of linear fits in 3 mice over 25 seizures.

To study oxidative metabolism near arteries during epileptic activity, 3 mice were recorded with measures at multiple locations near an artery that was close to the injection site. The percent pO_2_ value changes adjacent to the artery were significantly lower than values located farther away (an example shown in Fig 7A) despite some points being closer to the focus. Fig 7B shows a linear relationship between arterial perpendicular distance and percent of initial dip which indicates a contribution of the vascular anatomy to define the focus-surround regions. Sites that were farther from an artery, located in the capillary bed, elicited a larger decrease in tissue pO_2_ after onset. Extending data to the three mice, the slopes of these linear fit were combined over all seizures in Fig 7C, in all cases preserving the positive relationship. This data indicates that the vascular micro-environment contributes to oxygen consumption in the tissue during epileptic seizures.

### Correlation between initial dip and seizure duration

**Fig 8:**
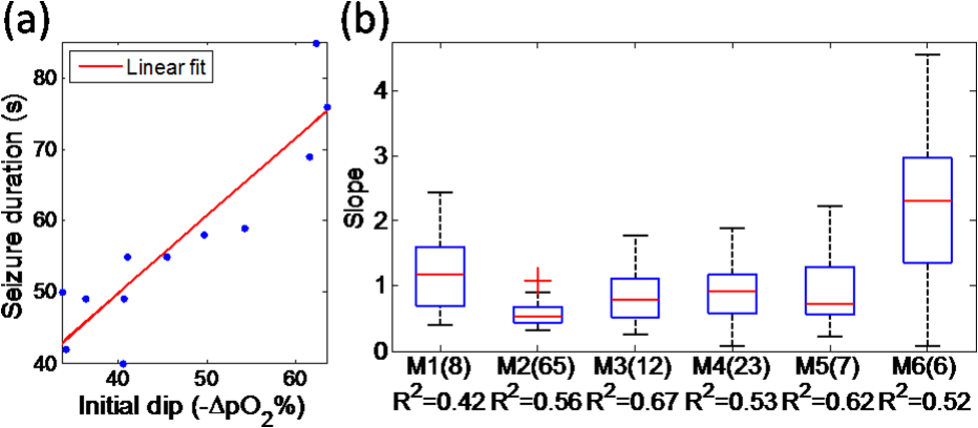
a) Correlation between initial dip (% change) and duration of epileptic activity. The line of linear fit was *y* = 0.74*x* − 4.35, R^2^ = 0.81 (b) Statistical distribution of the slopes for all mice. M1 was the name of mouse and number in the bracket was the number of seizure that was calculated. The outliers were plotted with red plus sign. The average of goodness of fit (R2) was listed for each mouse.

Because seizures had different durations, it was of interest to see if correlations between oxygenation and electrographic seizure duration were present. Linear regressions between the initial dip expressed as a percent change and seizure duration indeed showed significant correlations (an example shown in Fig 8A). This data suggest that long epileptic seizures may be accompanied by early increased oxygen consumption in the tissue. The slopes of fit between epileptic seizure duration and initial dip, distributed over spatial location measurements (shown in Fig 8B) over all seizures. Data in all mice show similar results: a positive moderate correlation was found between early metabolism in the interstitial space near the focus and duration of epileptic activities though the relationship had significant variability across the population.

## Discussion

The results of our investigation demonstrate that tissue oxygenation can be measured in the mouse cortex using a confocal phosphorescence lifetime measurements given appropriate excitation regime. With respect to brain studies, the key advantages of confocal lifetime system are its minimal disturbance of investigated tissue and the possibility to achieve high temporal and spatial resolution. The probe signal is independent of pH throughout the physiological range and is not affected by the presence of biological macromolecules [30]. However, each pO_***2***_ measurements required collection of 100 decays at each point to yield a reliable pO_***2***_ value, thus limiting the temporal resolution of the method. Moreover, light scattering by brain tissue limits our confocal pO_2_ measurements to tissue located up to 100μm deep near the cortical surface which may confound results investigating oxygen diffusion from arterioles. Combining lifetime-based pO_2_ monitoring with multiphoton excitation may help overcome some of these issues in future cerebral oxygenation investigations.

We have successfully used confocal lifetime system to investigate tissue pO_2_ in somatosensory cortex during normal state and epileptic activity induced by 4-AP. Our findings bring new evidence regarding tissue oxygen changes during epileptic activity, by characterizing the relationship between tissue oxygen changes and seizure duration.

### Tissue pO_2_ gradient near arteries in normoxia

Our work confirmed previous evidence that arteries are largely responsible for the heterogeneous oxygen distribution in the cortex. The observed variations in tissue pO_2_ values near brain arteries showed a drop in the pO_2_ values of points distant from an artery and located in the capillary bed during normoxia (Fig 3). Similar pO_2_ gradients were also found by several investigators employing different methods [27,32]. For instance, near the pial artery in the rat cortex, a similar tendency was reported by Sakadzic et al. [27] with two-photon phosphorescence lifetime measurements. In addition, several theoretical models have predicted that steep pO_2_ gradients arise in the vicinity of blood vessels [34,35]. Localized large tissue pO_2_ values suggests that arteries provide a major source of O_2_ to tissue [36] while our pO_2_ values of locations far away from arteries (above ~60μm) most likely rely on capillaries which are invisible from the cortical surface [37].

### Acute seizure activity leads to transient dip of pO_2_ near epileptic focus

Some controversy remains in the literature as to whether local cerebral blood flow increases are adequate to meet supranormal oxygen demands throughout the ictus. Although the inadequacy of cerebral blood flow in addressing oxygen demands has been demonstrated by some investigators, there is also growing evidence of inadequate oxygenation at the onset or throughout shorter duration epileptic events [38,39]. In this study, a clear transient decrease in tissue oxygenation at multiple locations near the injection site after onset was shown, indicating that the increased metabolism of oxygen overwhelms the ability of the brain to provide oxygenated blood by increasing cerebral blood flow during the seizure-like activity in and near the focus. This observation may be useful at predicting the location of seizures. A significant increase in tissue oxygen consumption at multiple locations close to the focus was observed in the somatosensory cortex when mice had seizure-like activity (Fig 6C). Moreover, farther away from the focus there was increase in tissue oxygen with respect to baseline.

Previous literature using oxygen microelectrodes documented a transient dip in two locations: near the focus and an increase in the surround in epileptic rats during seizures [33]. In our present work, which focused on tissue oxygen changes at multiple locations (>2) during acute epileptiform events, we show a significant initial dip at multiple locations in our mice. With the ability of our system to gather spatial measures, our results indicate that the distance between the surround and the injection site (around 1.5mm) was a little smaller than what was measured in previous work (around 2mm) [33]. Whether the observed difference is due to changes in animal species, high metabolic demand at seizure locations near vessels remains to be investigated.

### The relationship between the initial dip and distance from arterioles

Exploiting the spatial measures, we investigated how tissue oxygen pressure changed at different points near a surface arteriole located in the focus region during epileptiform activity. Despite increased consumption in the focus, arteriolar O_2_ diffusion remained partially unaffected: a significant increase in the initial dip was observed with increased distance from the arteriole (see Fig 7) in three mice. These data are the first showing measurements of pO_2_ gradients in brain arterioles during epileptic activity confirming that the arteriolar wall played a significant role in oxygen exchange between blood and tissue [32]. Our microscopic assessments thus paint a more complex spatial picture of oxygen consumption during epileptic seizures since arteries take part in diffusional exchange of respiratory gases (mainly oxygen) to tissue. This is in contrast to previous studies showing uniform cerebral blood volume (CBV) and cerebral blood flow (CBF) increase in the focus during epileptic events [33,40] to supply oxygen to meet demands of neuronal activity. The vascular architecture thus generates a microscopic structure to the epileptic focus, as our data showing increased initial dips far away from arteries, in capillary beds, suggest. Our results further suggest that the increased CBF and CBV will supply more oxygen to the tissue near an artery, but may not meet the demands of oxygen metabolism far away from an artery. Potential associated tissue and neuronal damage is thus more likely to occur, microscopically, in areas far from these feeding arteries.

### The relationship between initial dip and seizure duration

Our data indicate that initial dips of greater amplitude are predictive of seizures of greater duration. Few study has investigated such a link between oxygen metabolism and seizure duration. However, some researchers observed that a biphasic deoxyhemoglobin (HbR) response to ictal events with an initial decrease in HbR followed by a longer increase in HbR measured by NIRS may be related to longer seizure duration [39]. These previous findings proposed that increased seizure duration could lead to increased oxidative metabolism. The etiology of tissue oxygen changes as the duration of seizures is increased remains unknown. The possibility is that longer seizures were induced by increased initial neuronal activities, which will lead to more decrease in tissue oxygenation [41,42]. Our study indicates that it might be possible to predict seizure duration from the initial dip amplitude.

### Limitations

While we limited the number of pulses and counts during recordings to diminish photoconsumptive effects, survey scans could lead to the production of singlet oxygen as some areas were subject to higher light intensity than required by our 3000 counts estimation during the calibration phase. To address this issue, our acquisitions were careful to limit light intensity during survey scans and a pause was done between survey scans and acquisitions presented here to insure tissue oxygen is replenished. Despite these steps, we cannot completely rule out the possibility of tissue damage during survey scans due to singlet oxygen generated by the probe. Furthermore, the elongated focus of the confocal setup failed to precisely assess distribution of oxygen in the depth axis, limiting conclusions on oxygen diffusion during seizure, this we hope to revisit using the recently developed two-photon technique. Finally, a potential bias in measured PO2 values during epileptiform discharges may occur due to a small increase in temperature at the epileptic foci. Given the small change in decay parameters measured for G4 with fractional temperature change, this was neglected in estimations above.

## Conclusion

We developed an imaging technique that provides absolute values of pO_2_ in the brain cortex by means of confocal phosphorescence lifetime microscopy. The technique was applied to study partial oxygen pressure changes in tissue during epileptic activity in mice. To our knowledge, this is the first report of direct measurements of tissue oxygen at multiple locations (more than 2 locations) during epilepsy. In our work, following 4-AP injection in the somatosensory cortex of mice, we observed significant changes of pO_2_ in tissue near the injection site, and investigated its changes along arteries and in the surround. This study supported the existence of an initial dip and characterized the spatial distribution of the initial dip around the focus and near pial arteries. In addition, we found a positive correlation between the early oxygen metabolism in tissue and the duration of seizures. With regards to clinical relevance, our observations may eventually help the cause of epileptic focus localization and elucidate the link relating seizure duration and initial dip amplitude.
